# Outcomes of 4 years of molecular genetic diagnosis on a panel of genes involved in premature aging syndromes, including laminopathies and related disorders

**DOI:** 10.1186/s13023-019-1189-z

**Published:** 2019-12-11

**Authors:** Maude Grelet, Véronique Blanck, Sabine Sigaudy, Nicole Philip, Fabienne Giuliano, Khaoula Khachnaoui, Godelieve Morel, Sarah Grotto, Julia Sophie, Céline Poirsier, James Lespinasse, Laurent Alric, Patrick Calvas, Gihane Chalhoub, Valérie Layet, Arnaud Molin, Cindy Colson, Luisa Marsili, Patrick Edery, Nicolas Lévy, Annachiara De Sandre-Giovannoli

**Affiliations:** 1 Department of Medical Genetics, Assistance Publique Hopitaux de Marseille, Marseille, France; 2 Aix Marseille Univ, INSERM, MMG, Marseille, France; 3 Medical Genetics Unit 2, L’Archet Hospital, Nice, France; 4 Hospices Civils de Lyon, Genetic Department and National HHT Reference Center, Femme-Mère-Enfants Hospital, F-69677 Bron, France; 5 Université Claude Bernard Lyon 1, F-69100 Villeurbanne, France; 6 Genetics Department, AP-HP, Robert-Debré University Hospital, Paris, France; 7 Department of Medical Genetics, CHU Toulouse, Purpan Hospital, 31059 Toulouse, France; 8 Department of Genetics, Reims University Hospital, Reims, France; 9 Department of Genetics, Centre Hospitalier de Chambéry- Hôtel-dieu, Chambery, France; 10 Internal Medicine, CHU Toulouse, Rangueil Hospital, Toulouse 3 University Hospital Center, Toulouse, France; 11 Internal Medecine, CHR Metz-Thionville, Thionville, France; 12 Department of Genetics, Le Havre Hospital, F76600 Le Havre, France; 13 Department of Genetics, CHU de Caen, Avenue de la Cote de Nacre, 14000 Caen, France; 14 Department of Clinical Genetics, Lille University Hospital, CHU, Lille, France; 15 CRB-TAC (Biological Ressource Center-Tissues, DNA, Cells), Assistance Publique Hopitaux de Marseille, Marseille, France

**Keywords:** Progeroid, Panel, Pathogenic,variant

## Abstract

**Background:**

Segmental progeroid syndromes are a heterogeneous group of rare and often severe genetic disorders that have been studied since the twentieth century. These progeroid syndromes are defined as segmental because only some of the features observed during natural aging are accelerated.

**Methods:**

Since 2015, the Molecular Genetics Laboratory in Marseille La Timone Hospital proposes molecular diagnosis of premature aging syndromes including laminopathies and related disorders upon NGS sequencing of a panel of 82 genes involved in these syndromes.

We analyzed the results obtained in 4 years on 66 patients issued from France and abroad.

**Results:**

Globally, pathogenic or likely pathogenic variants (ACMG class 5 or 4) were identified in about 1/4 of the cases; among these, 9 pathogenic variants were novel. On the other hand, the diagnostic yield of our panel was over 60% when the patients were addressed upon a nosologically specific clinical suspicion, excepted for connective tissue disorders, for which clinical diagnosis may be more challenging. Prenatal testing was proposed to 3 families. We additionally detected 16 variants of uncertain significance and reclassified 3 of them as benign upon segregation analysis in first degree relatives.

**Conclusions:**

High throughput sequencing using the Laminopathies/ Premature Aging disorders panel allowed molecular diagnosis of rare disorders associated with premature aging features and genetic counseling for families, representing an interesting first-level analysis before whole genome sequencing may be proposed, as a future second step, by the National high throughput sequencing platforms (“Medicine France Genomics 2025” Plan), in families without molecular diagnosis.

**Electronic supplementary material:**

The online version of this article (10.1186/s13023-019-1189-z) contains supplementary material, which is available to authorized users.

## Background

Segmental progeroid syndromes are a heterogeneous group of rare genetic disorders, which were clinically described in medical journals since the twentieth century. These syndromes are defined as segmental because only some of the aging features are accelerated [1]. Historically, and based on their main pathophysiological bases, three major groups of progeroid syndromes may be distinguished.

The first consists of syndromes due to alteration of DNA-repair genes. The RecQ family genes, encoding DNA helicases, are mainly involved in this group of syndromes [2, 3]. DNA helicases are enzymes involved in different DNA-repair processes and are referred to as guardians of the genome. They play important roles in genome stability and maintenance [4]. Several syndromes are associated to the RecQ genes’ family including Werner syndrome (WS), Bloom syndrome (BS) and Rothmund Thomson syndrome (RTS) [5–7], that are respectively associated with pathogenic variants in *RECQL2/WRN, RECQL3/BLM,* and *RECQL4* genes. These syndromes share autosomal recessive inheritance and an increased susceptibility to cancer. WS is an adult-onset progeria syndrome, whereas the first symptoms of BS and RTS appear in childhood. Cockayne syndrome is another autosomal recessive disorder caused by mutations of DNA-repair genes, the CS proteins. Pathogenic variants of *CSA/ERCC8* and *CSB/ERCC6* genes are responsible for the majority of cases [1, 8, 9].

The second group deals with proteins maintaining the integrity of the nuclear envelope. Lamins A/C and their protein partners are mainly involved in this group of disorders. In 2003, De Sandre-Giovannoli et al. and Eriksson et al. identified a synonymous point mutation of the *LMNA* gene (NM_170707.4: c.1824C > T; p.Gly608Gly) as causative of the disease in patients affected with Hutchinson Gilford Progeria Syndrome (HGPS) [10, 11]. This synonymous and apparently harmless heterozygous variant activates a cryptic donor splice site in *LMNA* pre-mRNAs, leading to the production of a prelamin A precursor that lacks the cleavage recognition site for the endoprotease ZMPSTE24. As a result, a truncated and permanently prenylated prelamin A isoform named progerin accumulates and exerts multiple toxic effects in cells’ nuclei [10–12]. Other *LMNA* mutations affecting prelamin A maturation result in more or less severe progeroid syndromes, called HGPS-like, depending essentially on the quantities of progerin/prelamin A isoforms produced [13]. Two other syndromes, restrictive dermopathy (RD), a perinatal lethal genodermatosis, and type B mandibuloacral dysplasia (MAD-B), a relatively milder progeroid syndrome characterized by skeletal, metabolic and cutaneous abnormalities and lipodystrophy, have also been associated to pathological accumulation of prelamin A. Recessive pathogenic variants in *ZMPSTE24* have mostly been described in these syndromes, broadening the spectrum of prelamin-A associated disorders [1, 14–19]. Furthermore, several atypical progeroid syndromes (APS) or atypical Werner syndrome (AWS) with clinical features overlapping with HGPS and other prelamin A-linked disorders have been associated to missense mutations in the *LMNA* gene, which are often private and are seen in only a single or few families [20–27].

The third group is composed of syndromes characterized by features of premature aging resulting from diverse pathophysiological processes. This group includes all the disorders listed in Additional file 1: Table S1 and not belonging to the 1st and 2nd major groups of progeroid syndromes. It also includes Ehlers Danlos syndromes (EDS), a clinically and genetically heterogeneous group of diseases that affect connective tissues. EDS are classified into 13 subtypes according to the inheritance pattern, phenotype and pathogenetic mechanisms [28] and some of its subtypes present with premature aging signs. Cutis Laxa (CL) syndromes presenting as well with progeroid features are also included in this third group [29].

**Fig. 1:**
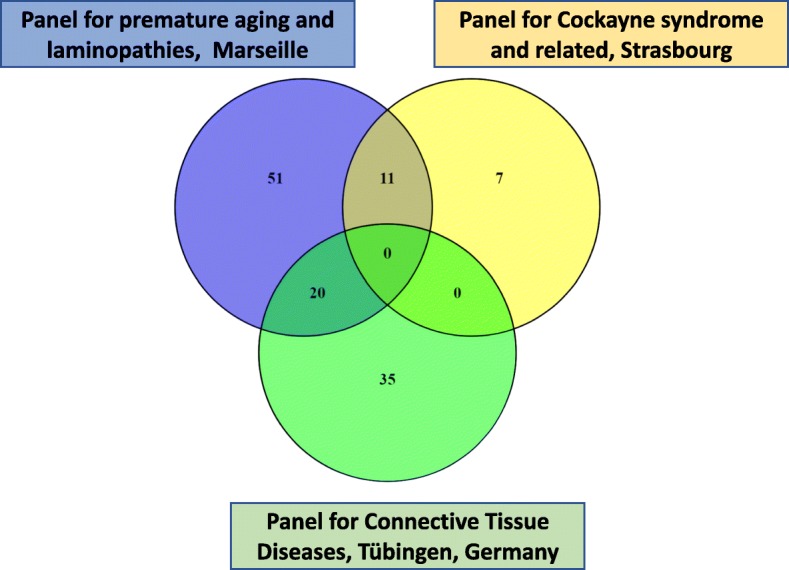
Venny diagrams showing the existing overlaps among available gene lists in a diagnostic setting according to Orphanet, available to test patients with premature aging disorders (source: https://www.orpha.net/consor/cgi-bin/index.php) and data available on the laboratories’ websites (https://www.cegat.de/en/diagnostics/diagnostic-panels/connective-tissue-diseases/; http://www.chru-strasbourg.fr/sites/default/files/u110/NER_18genes_190308_2.pdf). We compare our gene panel with a panel of 55 genes asssociated with connective tissue diseases in Tübingen, Germany (CeGaT GmbH) and a panel of 18 genes associated with Cockayne and related syndromes, in Strasbourg, France (CHU de Strasbourg, Hôpital Civil)

Since 2015, the molecular genetics laboratory in Marseille La Timone Hospital proposes molecular diagnosis of premature aging syndromes as well as laminopathies and related syndromes upon NGS (next generation sequencing) analysis of a panel of 82 genes involved in those disorders (Additional file 1: Table S1). To the best of our knowledge, this panel is unique in France (Fig. 1) and in Europe (https://www.orpha.net/consor/cgi-bin/ClinicalLabs.php?lng=FR).

We report herein the outcomes of 4 years of NGS molecular diagnosis in 66 index cases coming from France and abroad, affected with syndromes featuring premature aging, and attempt to provide a critical discussion of the results obtained.

## Materials and methods

### Next generation sequencing and mutation identification procedures

In a diagnostic setting, we performed the targeted analysis of 82 genes (Additional file 1: Table S1). The genes were included into the panel because of their association with laminopathies or with diseases including premature ageing features; they were chosen upon bibliographic search (PubMed) and using genetic/clinical databases (namely OMIM and Orphanet).

Since 2015, 66 index cases were subjected to the NGS analysis and included in this study. The criteria used to include patients in the molecular study were one of the following: (i) patients whose clinical diagnosis fitted with one of the disorders associated with a gene of the panel (Additional file 1: Table S1), whether it was a progeroid syndrome or not, but having a specific nosologic classification, (ii) patients for whom the prescribing physician suspected a disease related to premature aging, due the association of at least two premature aging signs, but without a specific nosologic classification.

DNA was extracted from peripheral blood samples by the Biological Resources Center CRB-TAC (with NF-S 86–900 and ISO-9001 V2015 certifications) of the Department of Medical Genetics.

We created a SureSelectXT 12-24 Mb library, custom 1032 gene panel in collaboration with Agilent Technologies (Santa Clara, California, USA), to be used for enrichment of targeted sequences for several molecular diagnosis indications in the laboratory. The coding regions and flanking intronic regions of the 1032 genes were enriched, in solution, using the SureSelect Target Enrichment System from Agilent (Santa Clara, California, USA), following the manufacturer recommendations. The Ion Proton platform (Thermo Fisher Scientific, USA) was used for high throughput sequencing. Then, raw data were converted to Fastq files and aligned to the reference sequence of the human genome (University of California Santa Cruz, hg19/GRCh37, (https://genome.ucsc.edu/), using the Torrent Suite software (Thermo Fisher). The same software was used to perform variant calling (germline_low_stringency_targetseq), with the following parameters (min_cov_each_strand: 0, min_variant_score: 10, min_allele_freq: 0.1, snp_min_coverage: 6 snp and indel; strand_bias: 0.98 snp and 0.85 indel). A variant calling format (VCF) file and binary alignment map and index (BAM/BAI) files were then obtained and used for variant annotation using the in-house software VarAFT (Variant Annotation and Filtration Tool, https://varaft.eu/) [30]). The list of genes to be analyzed for each patient was also selected using dedicated bedfiles by VarAFT. Sequence reads and variants’ visualization was performed using IGV (https://software.broadinstitute.org/software/igv/) [31].

The BEDTools-based VarAFT coverage module was used to compute breadth and depth of coverage data using BAM files for the selected gene list, producing a coverage report. Nucleotidic positions were interpreted only over a cutoff of 20X reads depth.

Variants whose allele frequencies were > 1% in GnomAD (https://gnomad.broadinstitute.org) and 1000 genomes (http://www.internationalgenome.org/) were removed, as well as deep intronic variants. The remaining variants were analyzed according to their genomic position and their predicted effects on RNAs and proteins. In order to study the pathogenicity of the variants, we used population databases such as gnomAD (https://gnomad.broadinstitute.org/), sequence databases such as ClinVar (https://www.ncbi.nlm.nih.gov/clinvar/), LOVD (http://www.lovd.nl/3.0/home), disease databases such as OMIM (https://www.omim.org/), integrated databases as ClinGen and in silico predictive algorithms such as Mutation Taster (http://www.mutationtaster.org/), Human Splicing Finder (http://www.umd.be/HSF3/) and UMD Predictor (http://umd-predictor.eu/), together with a review of the literature [32–34].

The variants were classified as “pathogenic”, “likely pathogenic”, “of uncertain significance”, “likely benign” or “benign” (respectively classes 5 to 1) according to the American College of Medical Genetics and Genomics (ACMG) classification, using Intervar (http://wintervar.wglab.org/), ClinVar (https://www.ncbi.nlm.nih.gov/clinvar/) and ClinGen (https://www.clinicalgenome.org/); the classification was manually adjusted using further ACMG criteria, whenever necessary, depending on the familial context and literature data [35]. In each case, the additional ACMG criteria allowing the reclassification of variants are given in Table 1.

**Table 1:** Pathogenic variants and VUS identified from 2015 to 2018 in our laboratory by NGS.

Patient	Age at referral	Indication	Gene	NM	Haplotype	cDNA change	Amino acid change	ACMG (default Intervar)	Details of Intervar	ACMG (Intervar reintepreted or manual-Maryland)	Additional criteria leading to the variant class change	ExAC	GnomAD	Mutation Taster	UMD-predictor	HSF	Literature
**P1**	**11m**	**CL 2B**	***PYCR1***	**NM_006907.3**	**hoz**	**c.616G>A**	**p.(GLy206Arg)**	**LP**	**PM1 PM2 PP3 PP5**	**P**	**PS1 PP1**	**1/23214 (4.308e-05)**	**2/186106 (1.07e-5)**	**D0.99**	**P 100**	**Probably no impact on splicing**	**Kretz et al. 2011; Reversade et al. 2009**
**P2**	**26y**	**RTS**	***RECQL4***	**NM_004260.3**	**comp.het**	**c.2263C>T**	**p.(Arg755Trp)**	**VUS**	**PM1 PM2 PP3 BP1**	**LP**	**PM3**	**NA**	**1/240964 (4.15e-6)**	**NA**	**NA**	**Potential alteration of splicing**	**THIS REPORT**
						**c.2415_2419dup**	**p.(Arg807fs113Ter)**	**NA**		**P**	**PM2 PVS1 PP3**	**NA**	**NA**	**NA**	**NA**	**Potential alteration of splicing**	**THIS REPORT**
**P3**	**9y2m**	**CL**	***ALDH18A1***	**NM_002860.4**	**het**	**c.413G>A**	**p.(Arg138Gln)**	**LP**	**PM1 PM2 PP2 PP3 PP5**	**P**	**PS1**	**NA**	**NA**	**D0.999**	**P 84**	**Potential alteration of splicing**	**Fischer-Zirnsak et al. 2015**
**P4**	**19y**	**BOFS**	***TFAP2A***	**NM_003220.3**	**het**	**c.710G>A**	**p.(Arg237Gln)**	**LP**	**PM1 PM2 PP2 PP3 PP5**	**P**	**PS1**	**NA**	**NA**	**D0.999**	**P 87**	**Potential alteration of splicing**	**Reiber et al. 2010 and p.(Arg237Gly) in Milunsky et al., 2010**
**P5**	**6m**	**CL**	***GORAB***	**NM_152281.2**	**comp.het**	**c.546A>T**	**p.(Glu182Asp)**	**VUS**	**PM2**	**LP**	**PM3 PP3 PP4**	**NA**	**NA**	**D0.999**	**PP72**	**Potential alteration of splicing**	**THIS REPORT**
						**c.859C>T**	**p.(Arg287Ter)**	**VUS**	**PM2 PP3 PP5**	**P**	**PVS1 PM3**	**2/121244 (1.65e-05)**	**3/251106 (1.19e-5)**	**D1**	**P100**	**Probably no impact on splicing**	**THIS REPORT**
**P6**	**8m**	**RTS**	***RECQL4***	**NM_004260.3**	**comp.het**	**c.1573delT**	**p.Cys525AlafsTer33**	**NA**		**P**	**PVS1 PM2 PM3 PP5**	**27/104602 (2.581e-04)**	**69/276484 (2.5e-4)**	**NA**	**NA**	**Potential alteration of splicing**	**Kitao et al. 1999; Siitonen et al. 2009**
						**c.2269C>T**	**p.(Gln757Ter)**	**P**	**PVS1 PM2 PP3 PP5**	**P**	**/**	**9/113308 (7.943e-05)**	**32/271122 (1.18e-4)**	**NA**	**NA**	**Potential alteration of splicing**	**Pujol et al.2000; Wang et al. 2003;Siitonen et al. 2009**
**P7●**	**19y**	**Cockayne Sd**	***ERCC6***	**NM_000124.4**	**het**	**c.2291T>C**	**p.(Leu764Ser)**	**VUS**	**PM1 PM2 PP3 BP1**	**LP**	**PM3 (deletion of the other allele)**	**NA**	**NA**	**D0.999**	**P90**	**Probably no impact on splicing**	**THIS REPORT**
**P8**	**41y**	**Werner Sd**	***WRN***	**NM_000553.5**	**comp.het**	**c.2313T>A**	**p.(Cys771Ter)**	**P**	**PVS1 PM2 PP3**	**P**	**/**	**NA**	**1/251244 (3.98e-6)**	**D1**	**P100**	**Probably no impact on splicing**	**THIS REPORT**
						**c.2665C>T**	**p.(Arg889Ter)**	**P**	**PVS1 PM2 PP3 PP5**	**P**	**/**	**4/120718 (3.314e-05)**	**6/251074 (2.39e-5)**	**D1**	**P100**	**Potential alteration of splicing**	http://www.pathology.washington.edu/research/werner/database/
**P9**	**43y**	**EDS**	***COL5A1***	**NM_000093.4**	**het**	**c.1884_1891del**	**p.(Asp629Phefs16Ter)**	**NA**		**LP**	**PVS1 PM2**	**NA**	**NA**	**NA**	**NA**	**Potential alteration of splicing**	**THIS REPORT**
**P10**	**24y**	**SHORT Sd**	***PIK3R1***	**NM_181523.2**	**het**	**c.1945C>T**	**p.(Arg649Trp)**	**LP**	**PM1 PM2 PP3 PP5**	**P**	**PS1 PP2**	**NA**	**NA**	**D0.999**	**P93**	**Potential alteration of splicing**	**Dyment et al. 2013**
**P11**	**63y**	**UPS**	***LMNA***	**NM_170707.4**	**het**	**c.1003C>T**	**p.(Arg335Trp)**	**VUS**	**PM2 PP3 PP5**	**P**	**PS1 PM1**	**NA**	**NA**	**D 0.999**	**P96**	**Probably no impact on splicing**	**Zaragova et al. 2017; Lambert et al., 2018**
**P12**	**37y**	**Buschke Ollendorf Sd**	***LEMD3***	**NM_014319.4**	**het**	**c.1323C>A**	**p.(Tyr441Ter)**	**VUS**	**PM2 PP3**	**P**	**PVS1 PS1 PP5**	**NA**	**NA**	**D1**	**P100**	**Potential alteration of splicing**	**Hellemans et al. 2006**
**P13**	**2m**	**CL**	***ALDH18A1***	**NM_002860.3**	**hoz**	**c.1499G>T**	**p.(Gly500Val)**	**LP**	**PM1 PM2 PP2 PP3**	**P**	**PP1 PM3**	**NA**	**0/239782 (0)**	**D0.99**	**P90**	**Potential alteration of splicing**	**THIS REPORT**
**P14**	**39y**	**EDS**	***COL5A1***	**NM_000093.4**	**comp.het**	**c.4030C>T**	**p.(Pro1344Ser)**	**VUS**	**PM2 PP3 BP1**	**VUS**	**/**	**NA**	**NA**	**D 0.999**	**P96**	**Potential alteration of splicing**	**THIS REPORT**
						**c.2374C>T**	**p.(Arg792Ter)**	**P**	**PVS1 PM2 PP3 PP5**	**P**	**/**	**NA**	**NA**	**D1**	**P100**	**Potential alteration of splicing**	**Wenstrup et al. 2010**
**P15**	**36y**	**Brittle cornea**	***PRDM5***	**NM_018699.2**	**hoz**	**c.1036C>T**	**p.(Arg346Ter)**	**P**	**PVS1 PM2 PP3**	**P**	**/**	**3/119938 (2.501e-05)**	**3/244748 (1.23e-5)**	**D1**	**P100**	**Potential alteration of splicing**	**THIS REPORT**
**P16**	**6y7m**	**Oculoden-todigital dysplasia**	***GJA1***	**NM_000165.5**	**het**	**c.287T>A**	**p.(Val96Glu)**	**LP**	**PM1 PM2 PP2 PP3**	**P**	**PS1 PP5**	**NA**	**NA**	**D0.99**	**P100**	**NA**	**Wiest et al. 2006**
P17	41y	EDS	*LMNA*	NM_005572.3 (Lamin C isoform)	het	c.1715G>A	p.(Arg572His)	VUS	PM2	VUS	/	NA	0/149540 (0)	Polym 0.992	Prob Polym	Probably no impact on splicing	THIS REPORT
P18	59y	Werner Sd	*LMNA*	NM_170707.4	het	c.1016C>T	p.(Ala339Val)	VUS	PM2 PP3	VUS	/	NA	NA	D0.999	P84	Potential alteration of splicing	THIS REPORT
P19	4m	UPS	*RECQL4*	NM_004260.3	hoz	c.2756-8G>T	p.?	NA		VUS	/	17/107602 (1.58e-04)	59/276606 (2.13e-4)	NA	NA	Probably no impact on splicing	THIS REPORT
P20	68y	EDS	*ALDH18A1*	NM_002860.4	het	c.1597G>A	p.(Val533Met)	VUS	PM1 PP2	VUS	/	NA	3/178100 (1.68e-5)	Polym 0,960	PP69	Probably no impact on splicing	THIS REPORT
P21	47y	EDS	*COL1A2*	NM_000089.3	het	c.798A>C	p.(Glu266Asp)	VUS	PM2	VUS	/	NA	2/282868 (7.07e-6)	D0,999	Polym45	Potential alteration of splicing	THIS REPORT
P22	20y	UPS	*SYNE1*	NM_182961.4	het	c.21209T>G	p.(Leu7070Trp)	VUS	BP1	VUS	/	NA	NA	Polym 0,890	P78	Potential alteration of splicing	THIS REPORT
P23^a^	21m	UPS	*ATR*	NM_001184.4	het	c.4750A>C	p.(Met1584Leu)	VUS	PM2	B	lack of segregation with the disease in the family	NA	3/251454 (1.19e-5)	D0,999	PP72	Potential alteration of splicing	THIS REPORT
P24^a^	17y	EDS	*COL1A1*	NM_000088.3	het	c.310G>A	p.(Asp104Asn)	VUS	PM2	B	lack of segregation with the disease in the family	NA	NA	Polym0,999	Polym33	Potential alteration of splicing	THIS REPORT
P25	57y	UPS	*COL5A2*	NM_000393.5	het	c.463C>T	p.(Arg155Cys)	VUS	PP3	VUS	/	1/49686 (2.013e-5)	7/209120 (3.35e-5)	D0,999	P100	Potential alteration of splicing	THIS REPORT
			*SYNE2*	NM_182914.2	het	c.20632_20634delinsGAA	p.(Ser6878Glu)	NA		VUS	/	NA	NA	NA	NA	Potential alteration of splicing	THIS REPORT
P26^a^	65y	UPS	*ATR*	NM_001184.4	hoz	c.7701A>G	p.(Lys2567Lys)	VUS	BP4 BP7	B	lack of segregation with the disease in the family	NA	NA	D1	P77	Potential alteration of splicing	THIS REPORT
P27	21y	UPS	*ELN*	NM_001278913.1	het	c.1063_1086del	p.(355_362del)	NA		VUS	/	NA	NA	NA	NA	NA	THIS REPORT
P28	28y	EDS	*TNXB*	NM_019105.7	het	c.747C>T	p.(Cys249Cys)	LB	PM2 BP4 BP7	VUS	/	1/102780 (9.73e-06)	1/245798 (4.07e-6)	D1	Polym 11	Potential alteration of splicing	THIS REPORT
P29	57y	UPS	*FBN1*	NM_000138.4	het	c.5347A>T	p.(Ile1783Phe)	VUS		VUS	/	NA	NA	D0,999	P78	Potential alteration of splicing	THIS REPORT
P30	19y	EDS	*ACAN*	NM_013227.3	het	c.7205G>A	p.(Arg2402His)	VUS	PM1 PM2	VUS	/	8/119916 (6.671e-05)	20/248986 (8.03e-5)	D0,999	P87	Probably no impact on splicing	THIS REPORT

Pathogenic variants are shown in bold, VUS are in plain text.^a^the variant has been searched in family, allowing us to reclassify a VUS into a benign variant, P23: the variant was absent from affected mother, P24 and P26: the variant was present in unaffected first degree relatives ; P7● also carried an heterozygous deletion of ER*CC6 . y*years old,*m*months old,*Sd*syndrome,*RTS*Rothmund Thomson Syndrome,*BOFS*Branchiooculofacial syndrome,*CL*Cutis laxa,*EDS*Ehlers Danlos Syndrome,*UPS*Unspecified Progeroid syndrome,*het*heterozygous,*comp.het*compound heterozygous,*hoz*homozygous,*P*pathogenic,*LP*likely pathogenic,*VUS*variant of unknown significance,*LB*likely benign,*B*benign,*NA*not availabke,*D*disease causing,*Polym*polymorphism,*P*pathogenic,*PP*probably pathogenic. Intervar:http://wintervar.wglab.org/; Maryland:http://www.medschool.umaryland.edu/Genetic_Variant_Interpretation_Tool1.html/

Only the variants of classes 5 to 3 were reported in molecular genetics diagnostic reports after Sanger sequencing confirmation. Genes were named following the Hugo gene nomenclature committee guidelines (https://www.genenames.org/); DNA mutations and predicted protein changes were named following the HGVS nomenclature guidelines (http://www.hgvs.org/mutnomen/).

### Sanger sequencing for the validation of variants

We designed primers for PCR amplification using the Primer3 software (http://bioinfo.ut.ee/primer3/) in order to amplify the gene’s exon. Briefly, Sanger sequencing was performed as follows: purification of PCR products was performed according to the manufacturer’s instructions and both strands were sequenced using the Big Dye Terminator V.1.1 Cycle Sequencing Kit (Applied Biosystems). Sequence reactions were purified on Sephadex G50 (Amersham Pharmacia Biotech, Foster City, California, USA). We performed capillary electrophoresis on a Genetic Analyzer ABI3500XL (Life Technologies, USA). Electropherograms were analyzed on the Sequence Analysis Software V.5.2 (Applied Biosystems) and aligned and interpreted relative to the reference sequence using Sequencher V.5.4.6 (Gene Codes Corporation).

## Results

**Fig. 2:**
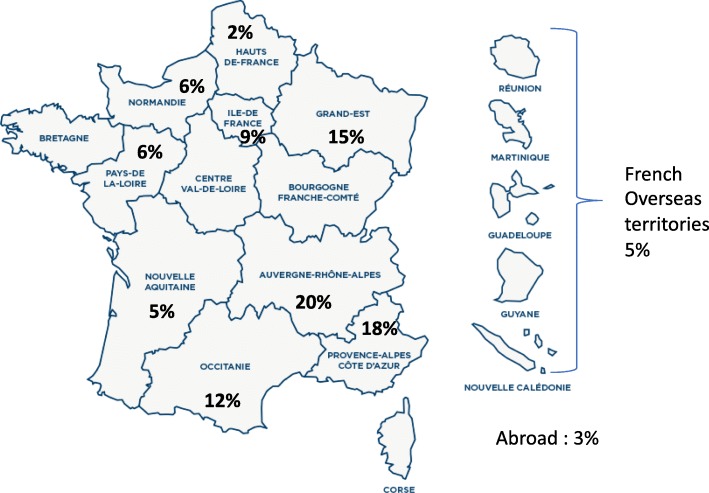
Map of France depicting the origin of index cases

Since 2015, we analyzed 66 patients from all over France and abroad on a panel of genes involved in premature aging syndromes. As depicted in Fig. 1, 50% of the patients were addressed from the southern regions of France and only 3% from abroad (Fig. 2). To the best of our knowledge, this kind of gene panel largely exploring premature aging syndromes and laminopathies is unique in France and Europe (https://www.orpha.net/consor/cgi-bin/index.php). Some overlap exists with other gene panels, specifically exploring sets of genes involved in particular diseases such as for example connective tissue disorders or Cockayne and related syndromes (Fig. 1).

The average coverage obtained at 20X on the list of genes and corresponding reference transcripts we describe (Additional file 1: Table S1) was 93% (248,228/267,610 nucleotides covered at 20X +/− 10,237 mean nucleotides, i.e. +/− 4% mean SD).

The molecular analysis allowed to identify 20 pathogenic or likely pathogenic variants in 16 patients according to ACMG, using Intervar (http://wintervar.wglab.org/), literature and databases, as described above (Table 1). Whenever necessary, the clinical interpretation of sequence variants was adjusted manually according to the literature, genetic databases and patients’ clinical data.

**Fig. 3:**
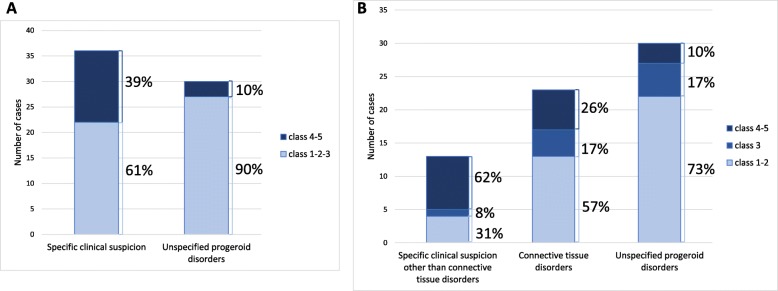
**a** and **b** Total numberof variants identified according to the clinical indication for analysis and corresponding ACMG classes. **a** the cohort is divided in two groups upon the specificity of the clinical suspicion, **b**: the “specific clinical suspicion” group was divided in two: “specific clinical suspicion other than connective tissue disorders” and “connective tissue disorders”

Globally, class 1–2 (benign and likely benign) variants were identified in 59% of the patients, class 3 variants (VUS, variant of unknown significance) in 15% and class 5 and 4 variants (pathogenic or likely pathogenic, respectively) in 26% of the patients (about 1/4). In order to analyze the molecular diagnostic yield with respect to the nosologic specificity of the clinical suspicion, we divided the index cases in two, then in three groups according to the clinical diagnosis/indication for NGS: respectively “specific clinical suspicion and unspecified progeroid disorders” then “specific clinical suspicion other than connective tissue disorders, connective tissue disorders (namely including CL and EDS) and unspecified progeroid disorders” (Fig. 3a, b). As expected, when considering the first group of patients with “specific clinical suspicion” we obtained a 39% diagnostic yield for ACMG classes 4 and 5 variants (likely pathogenic or pathogenic), compared to the “unspecified progeroid disorders” for which the same types of variants were retrieved only in 10% of the patients. When we further divided the first group in two, i.e. “specific clinical suspicion other than connective tissue disorders and connective tissue disorders”, we obtained a much higher diagnostic yield (62%) with ACMG classes 4 and 5 variants for patients with a nosologically defined clinical syndrome vs only 26% for connective tissue disorders (Fig. 3b). In the same graph, we can see that the detection rate of class 3 variants was the same for the two subgroups.

Table 1 compiles the 36 variants observed in our cohort from class 3 to 5; 9 of the 20 variants belonging to classes 4 and 5 are described in this report for the first time (Table 1, last column "THIS REPORT").

**Fig. 4:**
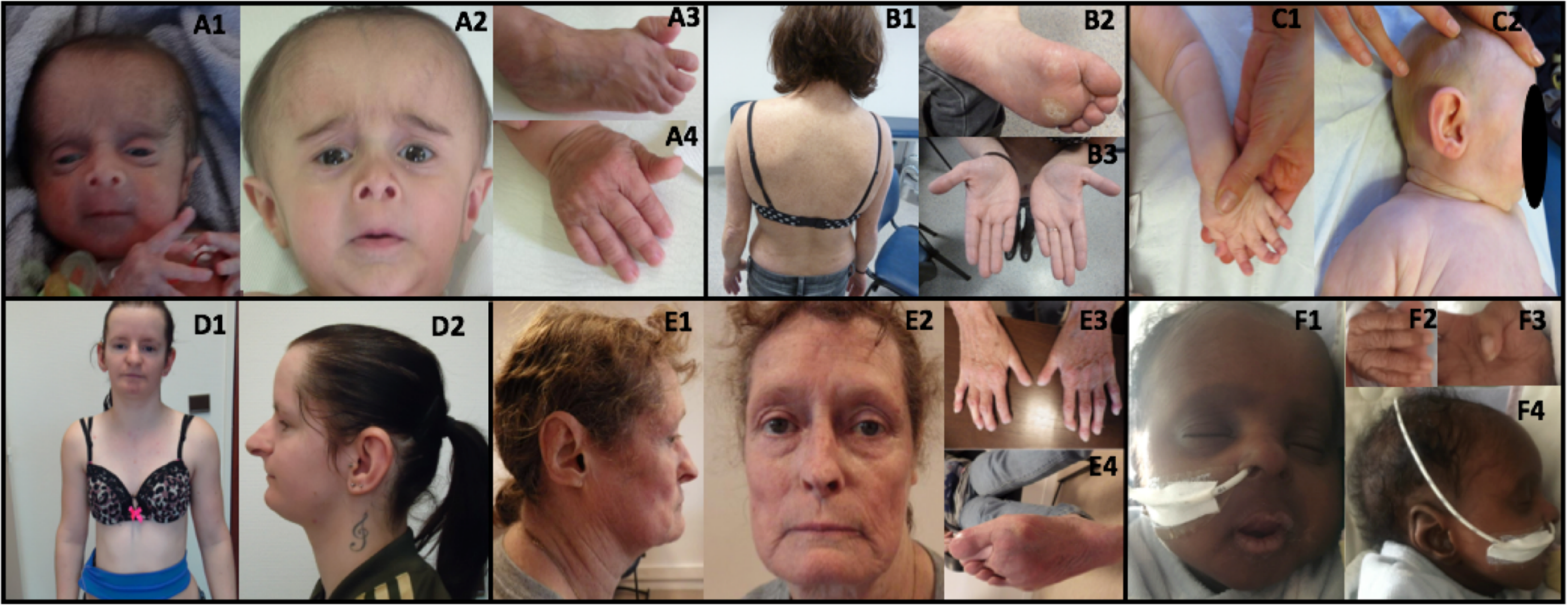
Pictures of patients P1, P2, P5, P10, P11, P13. A1–A4: Patient P1, on A1 ten days after birth and on A2–A4 at the age of 11 months, affected with De Barsy syndrome (*PYCR1*: homozygous c.616G>A); B1–B3 : Patient P2 affected with Rothmund-Thomson syndrome at the age of 26 (*RECQL4*: c.2263C>T and c.2415_2419dup) ; C1, C2: Patient P5 affected with Geroderma Osteodysplastica at the age of 5 months (*GORAB*: c.546A>T and c.859C>T); D1, D2: Patient P10 affected with SHORT syndrome (*PIK3R1*: c.1945C>T) at the age of 23 ; E1–E4 : Patient P11 at the age of 63, affected with a progeroid laminopathy (*LMNA*: c.1003C>T); F1–F4 : Patient P13 affected with cutis laxa (*ALDH18A1*: homozygous c.1499G>T) at the age of 1 month

In the group of nosologically defined, suspected clinical syndromes, we identified compound heterozygous pathogenic variants of RecQ family genes for three patients: *RECQL4* in two patients (P2 and P6) presenting with RTS and *WRN/RECQL2* in one patient with WS (P8). Both patients with RTS were female and had intrauterine and postnatal growth retardation, poikiloderma (collectively defining the following skin anomalies: reticulated hypo- and hyperpigmentation, punctate atrophy and telangiectasias) (Fig. 4B1-B3) and skeletal anomalies (fractures for patient P2 and decreased bone mineral density for patient P6). Patient P2 was older than patient P6, she developed a metastatic colon cancer and died at age 27. The pathogenic heterozygous variants found in patient P6 had already been described in a patient presenting with RTS [36–38], whereas the two pathogenic variants observed in patient P2 (c.2263C > T; p.(Arg755Trp) and c.2415_2419dup; p.(Arg807fs113Ter)) are described for the first time in this report. Patient P8 affected with WS presented with bilateral cataract, premature graying of scalp hair, alterations of the skin (thin skin and hyperpigmentation), soft tissue calcification, Achille’s tendinopathy, muscle atrophy and hypothyroidism at age 38. One of the pathogenic variants identified in the *WRN* gene (c.2313 T > A; p.(Cys771Ter)) is novel as well. Another patient (P7) was referred to our laboratory with clinical features of late onset Cockayne Syndrome. A deletion of 4,8 Mb encompassing the *ERCC6* gene was already identified by array CGH and we observed a missense variant in exon 12 on the other *ERCC6* allele (c.2291 T > C; p.(Leu764Ser)).

We identified already described pathogenic variants confirming the clinical diagnoses of SHORT Syndrome in patient P10, of Buschke-Ollendorf syndrome in patient P12, of Branchio-Oculo-Facial Syndrome (BOFS) in patient P4 and of Oculodentodigital Dysplasia in patient P16 (Table 1), respectively in the *PIK3R1, LEMD3/MAN1, TFAP2A* and *GJA1* genes [39–42]. As it can be seen in Fig. 4D1, D2, patient P10 affected with SHORT syndrome presented with short stature (below the 3rd SD), triangular face with prominent forehead, deep-set eyes, narrow tip of the nose, low-hanging columella and partial lipodystrophy at age 23. She also presented Axenfeld-Rieger anomaly, diabetes mellitus, hearing loss and ovarian cysts.

In the group of connective tissue disorders, we identified new compound heterozygous or homozygous class 4 or 5 variants in patients presenting with Cutis Laxa: *GORAB* (c.546A > T; p.(Glu182Asp)) and c.859C > T; p.(Arg287Ter)) for patient P5 and an homozygous novel variant in *ALDH18A1* (c.1499G > T; p.(Gly500Val)) for patient P13 (Table 1 and Fig. 4F1-F4). Patient P5 was evaluated in his first year of life: he showed joint laxity, bilateral congenital hip dislocation, fracture of the tibia, skin wrinkling, premature aged appearance of the face, deep set eyes, droopy cheeks and a pinched nose (Fig. 4C1, C2). His nonsense variant was inherited from the mother, who presented with a mild phenotype associating joint laxity and a pinched nose. *GORAB* compound heterozygous variants and the clinical features of patient P5 were compatible with Geroderma osteodyplastica (GO) or Autosomic Recessive Cutis Laxa type 2 (ARCL2) diagnoses. Patient P13 was the first child of a consanguineous couple from Senegal; IUGR (intrauterine growth retardation) was observed during pregnancy. He was born prematurely with height and weight below the third centile and his head circumference was between the 5th and 10th percentile. Clinical examination at 1 month of life evidenced retarded postnatal growth (below the 3rd centile, as at birth), hypotonia, failure to thrive, large fontanelles, facial progeroid appearance and cutis laxa. He also presented stenosis of the aortic arch. *ALDH18A1* pathogenic variants are responsible of De Barsy syndrome, previously known as ARCL3A, defining a molecular diagnosis that was compatible with the clinical features of patient P13 [29]. Other pathogenic variants already described in the literature confirmed the diagnosis of CL for patients P1 and P3 (Table 1 and Fig. 4A1-A4 for patient P1) [43–45]. Patient P1 (Fig. 4A1-A4) presented with aged appearance, a prominent forehead, loss of adipose tissue, translucent and wrinkly skin with visible veins. He also showed hypotonia and prenatal and postnatal growth retardation with conserved head circumference. This variant had already been associated with autosomal recessive Cutis Laxa [43, 44]. As mentioned above, EDS is another clinically and genetically heterogeneous group of connective tissue disorders. We identified two heterozygous nonsense variants in *COL5A1* in two patients presenting with classical EDS: one of them (c.1884_1891del; p.(Asp629Phefs16Ter) is described in this report for the first time, in patient P9, while the other (c.2374C > T; p.(Arg792Ter) [46]) was observed in a compound heterozygous state with a novel missense VUS in the same gene: (c.4030C > T; p.(Pro1344Ser)) in patient P14 (Table 1). Brittle Cornea syndrome is classified as one of the EDS subtypes [28, 42, 47]. In our cohort, patient P15, carried a homozygous nonsense variant in *PRMD5* that was not reported before (c.1036C > T; p.(Arg346Ter), Table 1). Patient P15 was issued from a consanguineous union; at the age of 35 years, he presented with bilateral keratoconus, keratoglobus with bilateral corneal transplants, bilateral cataract surgery, blue sclerae, hearing loss, scoliosis and joint laxity.

We additionally observed 3 *LMNA* variants, of which one was considered as pathogenic based on previous publications (c.1003C > T; p.(Arg335Trp)) [48–54]. This variant was previously described in two patients presenting with acro-osteolysis and dilated cardiomyopathy (DCM), another with a cardiac phenotype, acro-osteolysis and hypertriglyceridemia and other patients presenting with isolated DCM [48–52]. As described in Lambert et al., [54] patient P11 presented with a severe progeroid phenotype including acro-osteolysis with painful articulations of hands and feet, she had a pace-maker for heart rhythm disturbances (atrial fibrillation and atrioventricular block) without DCM on echocardiograms and she reported that children laughed at her at school for her “aged face and skin” [54]. Patient P11, shown in Fig. 4E1-E4 at 63 years old, presented with a pinched nose, malar hypoplasia, and an emaciated aspect of the face with aged, thin and dyspigmented skin, brachydactyly due to osteolysis predominating on the first three distal phalanges of both hands, and xerosis of the skin.

We additionally identified 16 variants of unknown significance (VUS, ACMG class 3 variants). Among them, 3 were reclassified as benign upon segregation analysis on DNAs of first-degree relatives. In the remaining cases, we didn’t receive parental DNAs to help reclassify them. Almost all VUS were identified in patients referred to our laboratory for Ehlers Danlos syndrome or unspecified progeroid syndromes. Among the suspected EDS, at least two patients (P20 and P28) had hypermobile EDS for which no gene was identified yet [28].

As mentioned above, one VUS in *COL5A1* was associated with a pathogenic variant of the same gene in patient P14. The patient almost fulfilled the diagnostic criteria of Classical-Like EDS (clEDS) syndrome, i.e. as major criteria: skin hyperextensibility, general joint hypermobility (Beighton score: 8/9), spontaneous ecchymoses, autosomal recessive inheritance because she was born form a consanguineous couple and minor criteria: hand acrogeria with clinodactyly, pes planus, hallux valgus. However, she also presented atrophic scarring which is a major criterion for Classical EDS (cEDS) and is normally absent from clEDS. She had one brother with clinical signs of EDS. We did not identify pathogenic variants in *TNXB* (whose coding sequence NM_019105.6 was entirely covered by the genomic sequencing), but we observed one *COL5A1* already known pathogenic variant (c.2374C > T; p.(Arg792Ter)) and one novel VUS (c.4030C > T; p.(Pro1344Ser)) [46]. Compound heterozygosity of *COL5A1* has already been described in one family, with one missense *COL5A1* variant playing the role of a “modifier gene” [55, 56]. In order to determine the impact of the VUS, the analysis of its segregation in the patient’s family would be useful.

Finally, two unreported heterozygous VUS that we would like to report were identified in the *LMNA* gene. One of these VUS (c.1016C > T; p.(Ala339Val)) was identified in patient P18, a 59 years old man, who displayed a range of premature-aging symptoms: premature balding since his twenties, short stature (− 2.5 SD), low body weight (− 2.25 DS), atrophic skin, sparse eyebrows and eyelashes, prominent eyes, micrognathia, and sloping shoulders. He suffered from intracranial hemorrhage at age 47 due to a ruptured posterior inferior cerebellar artery aneurysm. He also presented with aortic valve calcification associated with mild aortic insufficiency, incomplete right bundle branch block, hypertension, hypertriglyceridemia, and recurrent bone fractures upon osteoporosis. His cognitive development was normal. He was the only child of unrelated parents and he was childless. None of his relatives presented with premature aging features.

The other VUS in the *LMNA* gene was detected in patient P17 who was addressed for a clinical phenotype evocative of cEDS. This variant was located in the 3’UTR region of Lamin A-encoding transcripts (NM_170707.4: c.1698 + 17G > A) and in the last codon of Lamin C-encoding transcripts (NM_005572.3) (c.1715G > A; p.(Arg572His)). Unfortunately, no segregation study could be performed in the patient’s first-degree relatives.

## Discussion

We report the outcomes of 4 years of molecular genetic diagnosis by high throughput sequencing on a cohort of 66 patients mostly affected with premature aging syndromes, either nosologically classified or not, using a panel of 82 genes. To the best of our knowledge, this panel is the only available in France and in Europe to offer a wide molecular genetics exploration of disorders including features of premature aging. Other panels, mostly involved in the molecular diagnosis of connective tissue disorders or more restrained nosologic entities among premature aging syndromes, partially share genes with the panel we present.

This panel allowed us to provide molecular genetics results for patients from all over France and, in a few cases, from French overseas territories or abroad. As expected, and as seen in Fig. 1, more than half of the patients’ samples received in Marseille La Timone Molecular Genetics Laboratory were issued from the southern part of France. This is due to the strong previous interactions established in other diagnostic contexts with other southern French University Hospitals, and their facilitation by the establishment of financial agreements among regional hospitals in France. Nonetheless, the other cases were addressed from the rest of French territories or abroad, probably due to the unique availability of a large molecular screening in this kind of rare syndromes where, in some cases, large clinical overlaps can be observed. Additionally, this may be explained by the long-lasting involvement of our medical team in translational research and diagnosis of premature aging disorders, namely linked to Lamins A/C [1, 10, 13, 27, 57–62].

While for the global cohort of patients (*n* = 66) the diagnostic yield (i.e. identification of pathogenic or likely pathogenic variants) was of about 1/4 (26%), by further dividing the cohort in two groups, depending on the strength of the clinical suspicion towards a nosologically defined progeroid syndrome vs. an unspecified progeroid disorder (UPS), we observed that the diagnostic yield largely relied on this parameter, with almost 40% resolved cases in the first category of patients. When those groups were further subdivided in three (“specified clinical suspicion other than connective tissue disorders”, “connective tissue disorders” and “unspecified progeroid disorders”), the diagnostic yields were respectively 62, 26 and 10%. Indeed, this allows to observe, as expected, that the lowest yield is obtained in UPS, but a low yield is also retrieved in connective tissue disorders. This group of patients represents about 1/3rd of the cases addressed to our laboratory, with a clinical diagnosis or suspicion of EDS (Fig. 3 and data not shown). The International EDS Consortium recently defined 13 EDS subtypes and listed 19 causal genes [28]. In our panel, we analyze more than 50% of them, including the most frequently mutated ones (11/19). In France, to date, there is no available diagnostic panel exploring the 19 genes. For example, the extended panel of Tübingen in Germany, studies 90% of these 19 genes (17/19). Our panel thus represents the most extensive offer in France for EDS molecular diagnosis to date but it will be further implemented to include 19/19 genes in a future version. On the other hand, the number of patients that we received for this clinical indication points to a probably uncovered diagnostic need in France, together with the fact that it’s often hard to have a precise clinical suspicion given the high phenotypic variability of the EDS subtypes as well as the clinical overlap among EDS subtypes and other connective tissue disorders, making it interesting to use a large panel including most of the genes involved in those disorders with features of premature aging. This was also the reason why, although no specific gene has been identified yet, patients with a clinical diagnosis of hypermobile EDS were accepted and included in the molecular analysis. Indeed, more widely, many syndromes that are associated with the 82 genes included in our panel have overlapping clinical features [28, 63]. We report in this work 20 pathogenic or likely pathogenic variants in different clinical indications, 9 of them being novel. Among these novel variants 4 were non-sense and 2 frameshifting. We detected novel variants and in some cases reclassified them as pathogenic or likely pathogenic according to ACMG criteria, relative to genetic databases, literature and patients’ clinical history (Table 1): for *RECQL4* c.2263C > T; p.(Arg755Trp) and c.2415_2419dup; p.(Arg807fs113Ter) variants in patient P2 presenting with clinical RTS, for *GORAB* c.546A > T; p.(Glu182Asp) and c.859C > T; p.(Arg287Ter) in patient P5 presenting with clinical CL. Indeed, even if most pathogenic variants of *GORAB (alias: SCYL1BP1)* responsible of GO are nonsense, homozygous missense variants in compound heterozygosity with null alleles have already been associated with GO in previous publications [64, 65].

We also reclassified the *ERCC6* (c.2291 T > C; p.(Leu764Ser)) variant in patient P7 carrying a deletion of *ERCC6* on the other allele and a clinical diagnosis of late onset Cockayne syndrome; of note, Ghai et al. described a patient affected with Cokayne syndrome carrying almost the same deletion as P7 associated to a splice variant [66]. For *ALDH18A1* the homozygous variant: c.1499G > T; p.(Gly500Val) was reclassified in patient P13 presenting with features of CL; for *COL5A1*, the (c.1884_1891del; p.(Asp629Phefs16Ter)) nonsense variant was reclassified in patient P14 presenting with classical EDS. Other novel variants already classified as pathogenic by Intervar, an ACMG variant classification tool, were observed in *PRDM5* (homozygous variant: c.1036C > T; p.(Arg346Ter)) in patient P15 presenting with Brittle Cornea syndrome and in *WRN* (c.2313 T > A; p.(Cys771Ter)) associated with another already described nonsense variant in patient P8 presenting with classical Werner syndrome.

Other previously described pathogenic variants were identified in patients affected with syndromes as diverse as SHORT, Buschke-Ollendorf syndrome, Branchio-Oculo-Facial Syndrome (BOFS), and Oculodentodigital Dysplasia.

Among the 16 VUS observed, 3 were re-classified as benign by studying their segregation in first degree relatives’ DNAs.

Interestingly, three variants were detected in *LMNA*: one known pathogenic variant and two novel VUS, whose pathogenicity could not be proven for the moment, due to the lack of segregation studies or functional in vitro analyses. One of the *LMNA* VUS (NM_170707.4: c.1016C > T; p.(Ala339Val)) was found in a male proband (patient P18) who presented with an adult-onset progeroid phenotype suggestive of Atypical Werner Syndrome (AWS), described in patients presenting with some clinical signs of WS and carrying heterozygous missense *LMNA* variants [20, 22, 67–69]. Furthermore, the *WRN* gene, whose coding sequence NM_000553.5 was entirely covered by the genomic sequencing, did not show any pathogenic or likely pathogenic variants. We consider the *LMNA* p.(Ala339Val) variant as likely responsible for the patient’s phenotype, although it doesn’t fulfill ACMG criteria for (likely) pathogenicity [35]. Indeed, Ala339 is a very conserved residue and the *LMNA* gene has only a few well-known polymorphic variants (SNPs), making every undescribed variant very suspect of being pathogenic, especially if compatible with previously described laminopathies. Moreover, it is located nearby other previously reported AWS mutations [22, 68, 69].

The other *LMNA* VUS we observed (NM_005572.3: c.1715G > A; p.(Arg572His)) affected only Lamin C-encoding transcripts, being otherwise located in the 3′ UTR region of Lamin A-encoding transcripts, in patient P17, who was addressed for a suspicion of cEDS. It would be very interesting to perform the variant segregation study in this patient’s first relatives since, to the best of our knowledge, no variant affecting uniquely Lamin C isoforms has ever been described in human. Indeed, most laminopathies, including atypical progeria syndromes [24, 26, 70–72], have been associated to missense mutations affecting both Lamin A and C isoforms, and the mutation of only Lamin C isoforms may be compatible with a relatively mild clinical phenotype.

The familial segregation of these VUS and eventual functional analyses will be important to determine whether or not they are major pathogenic factors at the origin of the clinical phenotypes of the patients or only possible phenotype modifiers.

Patient P11 carrying the heterozygous c.1003C > T; p.(Arg335Trp) pathogenic variant in *LMNA* (NM_170707.4), already described in [48–54], presented since a young age with a severe progeroid phenotype of acro-osteolysis and cutaneous premature aging associated with heart rhythm disturbances; by giving us the authorization of publishing her pictures, the patient allows the medical community to become more familiar with the spectrum of clinical phenotypes (ranging from isolated dilated cardiomyopathy to premature aging phenotypes) linked to this particular *LMNA* mutation, as already reported for other *LMNA* mutations.

Finally, the identification of pathogenic variants allowed us to offer genetic counselling and propose prenatal testing: four prenatal tests were performed in the three families with mutations in *GORAB*, *RECQL4* and *TFAP2A*.

## Conclusion

Globally, the NGS molecular exploration of 66 patients using a panel of 82 genes associated with premature aging syndromes allowed us to confirm or establish a final diagnosis in 26% (about 1/4th) of the cases, while segregation analysis of first-degree relatives would help to reclassify the VUS identified in about 15% of the cohort.

The identification of a molecular diagnosis for these rare but often severe disorders, allowed to provide genetic counseling to the families and to propose prenatal diagnosis, contributing to personalized genomic, medical healthcare.

In order to further improve our panel’s diagnostic yield, clinicians will need to be sensitized to the utility of completing the segregation analyses in the families with VUS and sending at once the trios DNAs (affected patient and parents) whenever possible; also, research projects including functional in vitro analysis on human biological samples may be designed in order to include patients with VUS if the segregation analysis is compatible with a likely pathogenic effect, in order to provide arguments for it and refine the molecular diagnosis. On the other hand, for patients for whom only ACMG classes 1 or 2 were observed (which represent overall, about 60% of the patients of our cohort) further studies in a research context may be proposed.

Alternatively, the French government has financed the first two National high throughput whole genome sequencing platforms for trios; indeed, analysis of both parents and child are well known to improve the diagnostic performance [73, 74]. These WGS platforms have been established in the context of the “Medicine France Genomics 2025” Plan (https://solidarites-sante.gouv.fr/systeme-de-sante-et-medico-social/recherche-et-innovation/france-genomique), aiming to sustain and improve molecular genetic diagnosis of patients affected with rare disorders and therapeutic follow up of patients affected with cancer in the context of genomic, personalized medicine, allowing to further integrate scientific advances into healthcare and to facilitate the access to innovation to all patients. These patients and their relatives may also be candidate for this second level screening in the next years.

## Additional file

Additional file 1: **Tables S1.** Alphabetical list of genes included in the NGS panel “premature ageing syndromes and laminopathies”. AD: automal dominant; AR: autosomal recessive; Mi: mitochondrial; XLR: X linked recessive; XLD: X linked dominant; Smu: somatic mutation; IC: isolated cases. (XLSX 33 kb)

## Data Availability

The excel tables contained in the main text and/or in the supporting materials section will be freely downloadable. Please contact the corresponding author for other data requests.

## Abbreviations

aCGH: array comparative genomic hybridization
ACMG: American College of Medical Genetics
APS: Atypical progeroid syndromes
AWS: Atypical Werner Syndrome
BOFS: Branchio-Oculo-Facial Syndrome
BS: Bloom syndrome
cEDS: Classical EDS
CL: Cutis Laxa
clEDS: Classical-Like EDS
CRB-TAC: Biological Resources Center, Tissues-DNA-Cells
DCM: Dilated Cardiomyopathy
DNA: Desoxyribonucleic acid
EDS: Ehlers Danlos Syndrome
HGPS: Hutchinson Gilford Progeria Syndrome
MAD-B: Type B mandibuloacral dysplasia
RD: Restrictive Dermopathy
RTS: Rothmund Thomson Syndrome
SHORT: Short stature, Hyperextensibility of joints, Ocular depression, Rieger anomaly, Teething delay
UPS: Unspecified Progeroid Disorder
VUS: Variant of Unknown Significance
WS: Werner syndrome
